# Analyzing solitary wave solutions of the nonlinear Murray equation for blood flow in vessels with non-uniform wall properties

**DOI:** 10.1038/s41598-024-61276-z

**Published:** 2024-05-08

**Authors:** Mustafa Inc, Shabbir Hussain, Ali Hasan Ali, Muhammad Sajid Iqbal, Romana Ashraf, Muhammad Akhtar Tarar, Muhammad Adnan

**Affiliations:** 1 Department of Mathematics, Science Faculty, Firat University, 23119 Elazig, Turkey; 2 Department of Mathematics and Statistics, University of Lahore, Lahore, Pakistan; 3 Institute of Mathematics, University of Debrecen, Pf. 400, 4002 Debrecen, Hungary; 4 Department of Mathematics, College of Education for Pure Sciences, University of Basrah, 61001 Basrah, Iraq; 5 Technical Engineering College, Al-Ayen University, 64001 Dhi Qar, Iraq; 6 Department of Business Management, Al-imam University College, 34011 Balad, Iraq; 7 School of Foundation Studies and Mathematics, Liverpool john Moores University (UK), Qatar Campus, 12253 Doha, Qatar; 8 Department of Humanities & Basic Science, Military College of Signals, NUST, Islamabad, Pakistan; 9 Civil Engineering Department, The University of Lahore, Lahore, Pakistan

**Keywords:** Solitary wave solutions, Nonlinear Murray equation, Modified extended direct algebraic method, Applied mathematics, Computational models, Mathematics and computing

## Abstract

Solitary wave solutions are of great interest to bio-mathematicians and other scientists because they provide a basic description of nonlinear phenomena with many practical applications. They provide a strong foundation for the development of novel biological and medical models and therapies because of their remarkable behavior and persistence. They have the potential to improve our comprehension of intricate biological systems and help us create novel therapeutic approaches, which is something that researchers are actively investigating. In this study, solitary wave solutions of the nonlinear Murray equation will be discovered using a modified extended direct algebraic method. These solutions represent a uniform variation in blood vessel shape and diameter that can be used to stimulate blood flow in patients with cardiovascular disease. These solutions are newly in the literature, and give researchers an important tool for grasping complex biological systems. To see how the solitary wave solutions behave, graphs are displayed using Matlab.

## Introduction

Nonlinear partial differential equations are mathematical models used to describe nonlinear biological phenomena that involve several variables and interactions. Some examples of nonlinear biological phenomena that can be modeled using nonlinear partial differential equations include, reaction-diffusion systems, tumor growth and population dynamics. In addition to reaction-diffusion systems are nonlinear partial differential equations that describe the spread of chemical reactions through a medium. They are used to simulate how patterns formation in biological systems, such as the growth of bacterial colonies, the growth of animal coats, and the propagation of waves. The dispersion of electrical signals in neurons or the propagation of sound waves in the inner ear are examples of wave propagation in biological systems that can be described using nonlinear partial differential equations. The formation and spread of tumors in the body can be modeled using tumor growth nonlinear partial differential equations, which incorporate factors like nutrition availability, cell division rates, and interactions with the surrounding tissue. A population dynamics nonlinear partial differential equations can be used to simulate the dynamics of biological populations by considering factors like competition, predation, and environmental influences.

The Murray equation is a mathematical equation that explains the optimal size and branching angles of blood vessels in biological organisms. It simply says that the cube of a blood vessel diameter is proportional to the flow rate through it. But occasionally, the rate of blood flow through a blood vessel deviates from this straightforward relationship, necessitating the use of a more complicated variant of the Murray equation, known as the nonlinear Murray equation. The nonlinear Murray equation takes into consideration factors like the non-Newtonian behavior of blood flow and the elasticity of blood vessel walls.

Depending on the particular parameters and assumptions employed in the model, the nonlinear Murray equation precise form may change. A power law link between flow rate and vessel diameter is included in some versions of the equation, while more complex functional forms are used in others. Usually, sophisticated mathematical methods are needed to solve the nonlinear Murray equation. In the field of bio mechanics, research is now being done on the nonlinear Murray equation and its nonlinear extensions. The study of the Murray equation and its nonlinear extensions is an active area of research in the field of bio mechanics. The development of new treatments for cardiovascular disorders and other problems that affect the body blood flow can be greatly influenced by understanding the principles underlying the formation and function of blood vessels^9^.

In some research, the nonlinear Murray equation simplified forms have been derived exact solutions or analytical approximations. For instance, based on assumptions about the geometry and flow characteristics of the system, West et al.^4^ developed an analytical approximation for the optimal branching angle of blood vessels in a simplified form of the Murray equation. The Murray equation^8^, which represents blood flow in a single vessel, has a one-dimensional form that Olufsen et al.^5^ derived an exact solutions. This solution allowed for the examination of the effects of various physical conditions on blood flow in the vessel and was based on a linearization of the nonlinear Murray equation. More recently, a number of studies have examined the behavior of the nonlinear Murray equation under various circumstances using numerical simulation and other computational methods. For instance, Zhang et al.^6^ study used a finite element method^7^ to examine the effects of various physical parameters on the structure and function of a complex network of blood vessels.

A theory that explains the connection between the diameter of blood vessels and the rate of blood flow through them is known as Murray’s law or principle. This theory, which takes into account the impact of fluid dynamics, geometry, and other factors on the blood flow, is mathematically expressed by the non-linear Murray equation.

1 $$\begin{aligned} R^3 = C\left( \frac{Q}{w}\right) ^{2}+ \left( D \frac{w}{L}\right) , \end{aligned}$$

where: *R* is the radius of the blood vessel, *Q* is the blood flow rate through the vessel, *w* is the blood viscosity, *L* is the length of the vessel, *C* and *D* are constants. We take into account the nonlinear reaction-diffusion equation with convection term, which has the following form^1–3^

2 $$\begin{aligned} {\frac{\partial u }{\partial t}} = A \left( u \right) {\frac{\partial ^{2} u}{\partial {x}^{2}}} +B \left( u \right) {\frac{\partial u }{\partial x}} + C \left( u \right) , \end{aligned}$$

where $u\left( x,t \right)$ is an unknown function, *A*(*u*) , *B*(*u*) and *C*(*u*) are arbitrary smooth functions. When $A(u) = 1$, $B(u) = \alpha _{1} u$ and $C(u) = \alpha _{2} u - \alpha _{3} u^{2}$ where $\alpha _{1}, \alpha _{2}$ and $\alpha _{3} \in \mathbb {R}$, then Eq. (2) becomes

1.3 $$\begin{aligned} {\frac{\partial u }{\partial t}} = {\frac{\partial ^{2} u}{\partial {x}^{2}}} +\alpha _{1}u {\frac{\partial u }{\partial x}} + \alpha _{2} u -\alpha _{3} u^{2}, \end{aligned}$$

which is advanced form of the nonlinear Murray equation.

## Algorithm for modified extended direct algebraic method

In this section, we introduce the algorithm of modified extended direct algebraic method^9–16^ which is also known as the modified extended $\tanh$-function method^17–23^,

We provide an overview of the key steps involved in this method, which is explained in the following steps:

Suppose we have the following nonlinear PDE

4 $$\begin{aligned} P(u,u_{t},u_{x},u_{tt},u_{xx},u_{x t},\cdots ) =0, \end{aligned}$$

where $u=u(x,t)$ is an unknown wave function, *P* is a polynomial in $u=u(x,t)$ and its various partial derivatives in which the highest order derivatives and nonlinear terms are involved.

**Step 1**. Using the following wave transformation for traveling wave solutions

5 $$\begin{aligned} u=U(\xi ), \quad \xi = x-c\,t. \end{aligned}$$

where *c* is the wave speed.

**Step 2**. Plugging Eq. (5) into Eq. (4) yields a nonlinear ordinary differential equation

6 $$\begin{aligned} O(U, U', U'',U''',\cdots )=0. \end{aligned}$$

**Step 3**. Let $U(\xi )$ be the next variable that can be written as a polynomial in $\delta (\xi )$

7 $$\begin{aligned} U(\xi ) =\beta _{{0}}+\sum _{i=1}^{N}\beta _{{i}} \delta ^{i} \left( \xi \right) +\lambda _{{i}} \delta ^{-i} \left( \xi \right) , \end{aligned}$$

where $\beta _{0}, \beta _{1}, \beta _{2}, \lambda _{1}, \lambda _{2}$ are unknown constants to be determined later, while $\delta ^{'}(\xi )$ satisfies the nonlinear ODE

8 $$\begin{aligned} \delta '=\sigma +\delta ^{2}, \end{aligned}$$

where $\sigma$ is arbitrary constant, $\delta '=\delta (\xi )$ and $\delta '= \frac{d\delta }{d\xi }$.

**Step 4**. The homogeneous balance between the highest order derivatives and the nonlinear terms found in Eq. (6) can be examined in order to determine the value of the **natural number** *N*^24,25^

Plugging Eq. (7) into Eq. (5) along with Eq. (8) will yield a system of algebraic equations with respect to $\beta _{i},\lambda _{i}$, and $\sigma$ where $i = 1,2,3,\cdots N$. because all the coefficients of $\delta ^{i}$ have to vanish, we can then find $\beta _{0}$, $\beta _{{i}}$, $\lambda _{{i}}$, $\sigma$, and *c*. Eq. (8) has the general solutions:

**Family-1. If** $\sigma <0$, **we have**

9 $$\begin{aligned} \delta \left( \xi \right) = {-\sqrt{-\sigma }\tanh \left( \sqrt{-\sigma }\xi \right) } \quad \texttt {or} \quad \delta (\xi ) = -\sqrt{-\sigma }\coth \left( \sqrt{-\sigma }\xi \right) . \end{aligned}$$

it depends on the initial conditions.

**Family-2. If** $\sigma >0$, **we have**

10 $$\begin{aligned} \delta (\xi ) = {\sqrt{\sigma }\tan \left( \sqrt{\sigma }\xi \right) } \quad \texttt {or} \quad \delta (\xi ) = -\sqrt{\sigma }\cot \left( \sqrt{\sigma }\xi \right) , \end{aligned}$$

it depends on the initial conditions.

**Family-3: If** $\sigma =0$, **we have**

11 $$\begin{aligned} \delta \left( \xi \right) = -\dfrac{1}{\xi }. \end{aligned}$$

## Application of modified extended tanh-function method

Consider the nonlinear Murray equation

12 $$\begin{aligned} u_{xx}-u_{t}+\alpha _{{1}}uu_{x}+\alpha _{ {2}}u-\alpha _{{3}}u^{2} =0. \end{aligned}$$

Making wave transformation

13 $$\begin{aligned} u \left( x,t \right) =Q \left( \xi \right) = Q \left( x - c\,t \right) ,\,\,\,\, \xi =x-{ ct}. \end{aligned}$$

Plugging Eq. (13) into Eq. (12), we have

14 $$\begin{aligned} Q'' + c\,Q'+ \alpha _{1} QQ'+ \alpha _{2} Q - \alpha _{3}Q^{2}=0. \end{aligned}$$

Balancing $Q''$ with $Q^2$ in Eq. (14) gives $N = 2$. So, using Eq. (7) we have

15 $$\begin{aligned} Q =\beta _{{0}}+\beta _{{1}}\delta +\beta _{{2}}\delta ^{2} +{\frac{\lambda _{{1}}}{\delta }} +{\frac{\lambda _{{2}}}{\delta ^{2}}}, \end{aligned}$$

wherein $\beta _{0},\, \, \beta _{1}\, \beta _{2}\,$ and $\, \lambda _{1}, \lambda _{2}$ are arbitrary constants. Plugging Eq. (15) along with Eq. (8) into Eq. (14) will provide these constants, as well as collecting all terms with the same power of $\delta ^{i}, i = 0, 1,\cdots , N$ and setting every coefficient equal to zero, hence the following collection of algebraic equations is obtained

16 $$\begin{aligned} {\left\{ \begin{array}{ll} 6\,\beta _{{2}}+3\,\alpha _{{1}}\beta _{{1}}\beta _{{2}}-\alpha _{{3}}{ \beta _{{2}}}^{2}=0,\\ 2\,c\beta _{{2}}+2\,\beta _{{1}}+2\,\alpha _{{1}}{\beta _{{2}}}^{2}\sigma - 2\,\alpha _{{3}}\beta _{{1}}\beta _{{2}}+\alpha _{{1}}{\beta _{{1}}}^{2}+2 \,\alpha _{{1}}\beta _{{0}}\beta _{{2}}=0,\\ 8\,\beta _{{2}}\sigma +\alpha _{{1}}\beta _{{0}}\beta _{{1}}+3\,\alpha _{{1} }\beta _{{1}}\beta _{{2}}\sigma +\alpha _{{2}}\beta _{{2}}+\alpha _{{1}} \lambda _{{1}}\beta _{{2}}-2\,\alpha _{{3}}\beta _{{0}}\beta _{{2}}+c\beta _ {{1}}-\alpha _{{3}}{\beta _{{1}}}^{2}=0,\\ 2\,\alpha _{{1}}\beta _{{0}}\beta _{{2}}\sigma +2\,\beta _{{1}}\sigma -2\, \alpha _{{3}}\lambda _{{1}}\beta _{{2}}-2\,\alpha _{{3}}\beta _{{0}}\beta _{ {1}}+2\,c\beta _{{2}}\sigma +\alpha _{{2}}\beta _{{1}}+\alpha _{{1}}{\beta _ {{1}}}^{2}\sigma =0,\\ c\beta _{{1}}\sigma +\alpha _{{1}}\beta _{{0}}\beta _{{1}}\sigma +\alpha _{{2 }}\beta _{{0}}-c\lambda _{{1}}-2\,\alpha _{{3}}\beta _{{1}}\lambda _{{1}}- \alpha _{{1}}\beta _{{0}}\lambda _{{1}}+2\,\beta _{{2}}{\sigma }^{2}+2\, \lambda _{{2}}-\alpha _{{3}}{\beta _{{0}}}^{2}+\alpha _{{1}}\lambda _{{1}} \beta _{{2}}\sigma -2\,\alpha _{{3}}\beta _{{2}}\lambda _{{2}}-\alpha _{{1}} \beta _{{1}}\lambda _{{2}}=0,\\ 2\,\sigma \,\lambda _{{1}}-2\,c\lambda _{{2}}-2\,\alpha _{{3}}\beta _{{0}} \lambda _{{1}}+\alpha _{{2}}\lambda _{{1}}-2\,\alpha _{{1}}\beta _{{0}} \lambda _{{2}}-2\,\alpha _{{3}}\beta _{{1}}\lambda _{{2}}-\alpha _{{1}}{ \lambda _{{1}}}^{2}=0,\\ -2\,\alpha _{{3}}\beta _{{0}}\lambda _{{2}}-c\lambda _{{1}}\sigma +\alpha _{ {2}}\lambda _{{2}}-3\,\alpha _{{1}}\lambda _{{1}}\lambda _{{2}}-\alpha _{{1 }}\beta _{{1}}\lambda _{{2}}\sigma +8\,\lambda _{{2}}\sigma -\alpha _{{3}}{ \lambda _{{1}}}^{2}-\alpha _{{1}}\beta _{{0}}\lambda _{{1}}\sigma =0,\\ 2\,{\sigma }^{2}\lambda _{{1}}-2\,c\lambda _{{2}}\sigma -2\,\alpha _{{1}}{ \lambda _{{2}}}^{2}-2\,\alpha _{{3}}\lambda _{{1}}\lambda _{{2}}-2\,\alpha _{{1}}\beta _{{0}}\lambda _{{2}}\sigma -\alpha _{{1}}{\lambda _{{1}}}^{2} \sigma =0,\\ -\alpha _{{3}}{\lambda _{{2}}}^{2}+6\,\lambda _{{2}}{\sigma }^{2}-3\, \alpha _{{1}}\lambda _{{1}}\lambda _{{2}}\sigma =0. \end{array}\right. } \end{aligned}$$

The following set of solutions are possible for solving the 16 using Maple.

**Case 1**. Let $\beta _{0}=0,\beta _{1}=0, \lambda _{{1}}=0,$ and $\beta _{{2}}, \lambda _{{2}},c,\sigma$ are free parameters

17 $$\begin{aligned} \beta _{{2}}={\frac{{6}}{\alpha _{{3}}}},\,\,\,\, \lambda _{{2}}=\,{\frac{{6\sigma }^{2}}{5\alpha _{{3}}}}, \,\,\, c=c. \end{aligned}$$

**Case 2**. Let $\beta _{0}=0,\beta _{1}=0,\beta _{2}=0,$ and $\lambda _{{1}},\lambda _{{2}},\sigma , c$ are free parameters

18 $$\begin{aligned} \lambda _{{1}}=\,{\frac{{12\sigma }^{2}}{\alpha _{{3}}c+6\,\alpha _{{1}} \sigma }},\,\,\,\,\, \lambda _{{2}}=\,{\frac{{6\sigma }^{2}c}{\alpha _{{3}}c+6\,\alpha _{{1}}\sigma }}, \,\,\,\,c=c. \end{aligned}$$

**Case 3**. Let $\beta _{0} = 0, \beta _{{1}}= 0$, $\lambda _{{2}}=0,$ and $\beta _{{2}} ,\lambda _{{1}}, c, \sigma$ are free parameters

19 $$\begin{aligned} \beta _{{2}}={\frac{{6}}{\alpha _{{3}}}}, \,\,\,\,\,\lambda _{{1}}=-{\frac{8\,\sigma +\alpha _{{2}}}{\alpha _{{1}}}},\, \,\, c=-\,{\frac{6\alpha _{{1}}\sigma }{\alpha _{{3}}}}. \end{aligned}$$

**Case 4**. Let $\lambda _{1}=0,\beta _{1}=0$ and $\beta _{{0}}, \beta _{{2}}, \lambda _{{2}}, c, \sigma ,$ are free parameters

20 $$\begin{aligned} \beta _{{0}}= \,{\frac{8\,\sigma +\alpha _{{2}}}{2\alpha _{{3}}}},\,\,\, \beta _{{2}}={\frac{{6}}{\alpha _{{3}}}}, \,\,\lambda _{{2}}=\,{\frac{{6\sigma }^{2}}{\alpha _{{3}}}},\,\,\, c=-\,{\frac{\alpha _{{1}} \left( 20\,\sigma +\alpha _{{2}} \right) }{2\alpha _{{3}}}}. \end{aligned}$$

**Case 5**. Let $\beta _{1}=0,\beta _{2}=0,\lambda _{{1}}=0,$ and $\beta _{0}, \lambda _{{2}}, c, \sigma$ are free parameters

21 $$\begin{aligned} \beta _{{0}}= \dfrac{1}{2}\,{\frac{ \alpha _{{2}}\pm \sqrt{{\alpha _{{2}}}^{2}+8\, \alpha _{{3}}\lambda _{{2}}}}{\alpha _{{3}}}},\,\,\,\, \lambda _{{2}}={\frac{{6\,\sigma }^{2}}{\alpha _{{3}}}},\,\,\,\, c=\dfrac{1}{2}\,{\frac{\alpha _{{1}} \left( \alpha _{{2}}+\sqrt{{\alpha _{{2}}} ^{2}+48\,{\sigma }^{2}} \right) }{\alpha _{{3}}}}. \end{aligned}$$

**The following solutions are the families of solitary wave solutions of Eq.** 12**for different cases**17, 18, 19, 20, 21:

**Case 1**. Plugging Eq. (17) along with Eq. (15) into Eq. (13), then solitary waves solutions of Eq. (12) can be expressed in form of following families:

**Family-1**. When $\sigma <0$, then the possible solutions are

22 $$\begin{aligned} u_{1}\left( x,t\right)= & {} \dfrac{1}{2}\,{\frac{ \alpha _{{2}}\pm \sqrt{{\alpha _{{2}}}^{2}+8\,\alpha _{{3}}\lambda _{{2}}}}{\alpha _{{3}}}} + \,{\frac{{6\,\sigma }^{2}}{\alpha _{{3}}}} \bigg ({-\sqrt{-\sigma }\tanh \left( \sqrt{-\sigma }\xi \right) } \bigg )^{-2}, \end{aligned}$$

23 $$\begin{aligned} u_{2}\left( x,t\right)= & {} {\frac{{6}}{\alpha _{{3}}}}\bigg ({-\sqrt{-\sigma }\coth \left( \sqrt{-\sigma }\xi \right) } \bigg )^{2} + {\frac{{6\,\sigma }^{2}}{\alpha _{{3}}}} \bigg ({-\sqrt{-\sigma }\coth \left( \sqrt{-\sigma }\xi \right) } \bigg )^{-2}, \end{aligned}$$

where $$\xi =x-{ ct}$$.

**Family-2**. When $\sigma >0$, then the possible solutions are

24 $$\begin{aligned} u_{3}\left( x,t\right)= & {} {\frac{{6}}{\alpha _{{3}}}}\bigg ( \sqrt{\sigma }\tan \left( \sqrt{\sigma }\xi \right) \bigg )^{2} + {\frac{{6\,\sigma }^{2}}{\alpha _{{3}}}} \bigg (\sqrt{\sigma }\tan \left( \sqrt{\sigma }\xi \right) \bigg )^{-2}, \end{aligned}$$

25 $$\begin{aligned} u_{4}\left( x,t\right)= & {} {\frac{{6}}{\alpha _{{3}}}}\bigg ( -\sqrt{\sigma }\cot \left( \sqrt{\sigma }\xi \right) \bigg )^{2} +{\frac{{6\,\sigma }^{2}}{\alpha _{{3}}}} \bigg (-\sqrt{\sigma }\cot \left( \sqrt{\sigma }\xi \right) \bigg )^{-2}, \end{aligned}$$

where $$\xi =x-{ ct}$$.

**Family-3**. When $\sigma = 0$, then the possible solutions are solutions

26 $$\begin{aligned} u_{5}\left( x,t\right) = {\frac{{6}}{\alpha _{{3}}}}(-\frac{1}{\xi })^2, \end{aligned}$$

where $$\xi =x-{ ct}$$.

**Case 2**. Plugging Eq. (18) along with Eq. (15) into Eq. (13), then solitary waves solutions of Eq. (12) can be expressed in form of following families:

**Family-1. When** $\sigma <0$, **then the possible solutions are **

27 $$\begin{aligned} u_{6}\left( x,t\right)= & {} \,{\frac{{12\sigma }^{2}}{\alpha _{{3}}c+6\,\alpha _{{1}} \sigma }} \bigg ({-\sqrt{-\sigma }\tanh \left( \sqrt{-\sigma }\xi \right) } \bigg )^{-1} +{\frac{{6\sigma }^{2}c}{\alpha _{{3}}c+6\,\alpha _{{1}}\sigma }} \bigg ({-\sqrt{-\sigma }\tanh \left( \sqrt{-\sigma }\xi \right) } \bigg )^{-2}, \end{aligned}$$

28 $$\begin{aligned} u_{7}\left( x,t\right)= & {} \,{\frac{{12\sigma }^{2}}{\alpha _{{3}}c+6\,\alpha _{{1}} \sigma }} \bigg ({-\sqrt{-\sigma }\coth \left( \sqrt{-\sigma }\xi \right) } \bigg )^{-1} +{\frac{{6\sigma }^{2}c}{\alpha _{{3}}c+6\,\alpha _{{1}}\sigma }} \bigg ({-\sqrt{-\sigma }\coth \left( \sqrt{-\sigma }\xi \right) } \bigg )^{-2}, \end{aligned}$$

where $$\xi =x-{ ct}$$.

**Family-2. When** $\sigma >0$, ** then the possible solutions are **

29 $$\begin{aligned} u_{8}\left( x,t\right)= & {} \,{\frac{{12\sigma }^{2}}{\alpha _{{3}}c+6\,\alpha _{{1}} \sigma }} \bigg ({\sqrt{\sigma }\tan \left( \sqrt{\sigma }\xi \right) } \bigg )^{-1} +{\frac{{6\sigma }^{2}c}{\alpha _{{3}}c+6\,\alpha _{{1}}\sigma }} \bigg ({\sqrt{\sigma }\tan \left( \sqrt{\sigma }\xi \right) } \bigg )^{-2}, \end{aligned}$$

30 $$\begin{aligned} u_{9}\left( x,t\right)= & {} \,{\frac{{12\sigma }^{2}}{\alpha _{{3}}c+6\,\alpha _{{1}} \sigma }} \bigg ({-\sqrt{\sigma }\cot \left( \sqrt{\sigma }\xi \right) } \bigg )^{-1} +{\frac{{6\sigma }^{2}c}{\alpha _{{3}}c+6\,\alpha _{{1}}\sigma }} \bigg ({-\sqrt{\sigma }\cot \left( \sqrt{\sigma }\xi \right) } \bigg )^{-2}, \end{aligned}$$

where $$\xi =x-{ ct}$$.

**Case 3**. Plugging Eq. (19) along with Eq. (15) into Eq. (13), then solitary waves solutions of Eq. (12) can be expressed in form of following families:

**Family-1. When** $\sigma <0$, ** then the possible solutions are **

31 $$\begin{aligned} u_{10}\left( x,t\right)= & {} -{\frac{8\,\sigma +\alpha _{{2}}}{\alpha _{{1}}}}\bigg ({-\sqrt{-\sigma }\tanh \left( \sqrt{-\sigma }\xi \right) } \bigg )^{-1} +{\frac{{6}}{\alpha _{{3}}}} \bigg ({-\sqrt{-\sigma }\tanh \left( \sqrt{-\sigma }\xi \right) } \bigg )^{2}, \end{aligned}$$

32 $$\begin{aligned} u_{11}\left( x,t\right)= & {} -{\frac{8\,\sigma +\alpha _{{2}}}{\alpha _{{1}}}}\bigg ({-\sqrt{-\sigma }\coth \left( \sqrt{-\sigma }\xi \right) } \bigg )^{-1} +{\frac{{6}}{\alpha _{{3}}}} \bigg ({-\sqrt{-\sigma }\coth \left( \sqrt{-\sigma }\xi \right) } \bigg )^{2}, \end{aligned}$$

where $$\xi =x-{ ct}$$.

**Family-2. When** $\sigma >0$, **then the possible solutions are **

33 $$\begin{aligned} u_{12}\left( x,t\right)= & {} -{\frac{8\,\sigma +\alpha _{{2}}}{\alpha _{{1}}}}\bigg ({\sqrt{\sigma }\tan \left( \sqrt{\sigma }\xi \right) } \bigg )^{-1} +{\frac{{6}}{\alpha _{{3}}}} \bigg ({\sqrt{\sigma }\tan \left( \sqrt{\sigma }\xi \right) } \bigg )^{2}, \end{aligned}$$

34 $$\begin{aligned} u_{13}\left( x,t\right)= & {} -{\frac{8\,\sigma +\alpha _{{2}}}{\alpha _{{1}}}}\bigg ({-\sqrt{\sigma }\cot \left( \sqrt{\sigma }\xi \right) } \bigg )^{-1} +{\frac{{6}}{\alpha _{{3}}}} \bigg ({-\sqrt{\sigma }\cot \left( \sqrt{\sigma }\xi \right) } \bigg )^{2}, \end{aligned}$$

where $$\xi =x-{ ct}$$.

**Case 4.** Plugging Eq. (20) along with Eq. (15) into Eq. (13), then solitary waves solutions of Eq. (12) can be expressed in form of following families:

**Family-1. When** $\sigma <0$, **then the possible solutions are **

35 $$\begin{aligned} u_{14}\left( x,t\right)= & {} {\frac{8\,\sigma +\alpha _{{2}}}{2\alpha _{{3}}}} + {\frac{{6}}{\alpha _{{3}}}} \bigg (-{\sqrt{-\sigma }\tanh \left( \sqrt{-\sigma }\xi \right) } \bigg )^{2} + {\frac{{6\sigma }^{2}}{\alpha _{{3}}}}\bigg ({-\sqrt{-\sigma }\tanh \left( \sqrt{-\sigma }\xi \right) } \bigg )^{-2}, \end{aligned}$$

36 $$\begin{aligned} u_{15}\left( x,t\right)= & {} {\frac{8\,\sigma +\alpha _{{2}}}{2\alpha _{{3}}}} + {\frac{{6}}{\alpha _{{3}}}} \bigg (-{\sqrt{-\sigma }\coth \left( \sqrt{-\sigma }\xi \right) } \bigg )^{2} + {\frac{{6\sigma }^{2}}{\alpha _{{3}}}}\bigg ({-\sqrt{-\sigma }\coth \left( \sqrt{-\sigma }\xi \right) } \bigg )^{-2}, \end{aligned}$$

where $$\xi =x-{ \left( -\,{\frac{\alpha _{{1}} \left( 20\,\sigma +\alpha _{{2}} \right) }{2\alpha _{{3}}}}\right) t}$$.

**Family-2. When** $\sigma >0$, **then the possible solutions are **

37 $$\begin{aligned} u_{16}\left( x,t\right)= & {} {\frac{8\,\sigma +\alpha _{{2}}}{2\alpha _{{3}}}} + {\frac{{6}}{\alpha _{{3}}}} \bigg ({\sqrt{\sigma }\tan \left( \sqrt{\sigma }\xi \right) } \bigg )^{2} + {\frac{{6\sigma }^{2}}{\alpha _{{3}}}}\bigg ({\sqrt{\sigma }\tan \left( \sqrt{\sigma }\xi \right) } \bigg )^{-2}, \end{aligned}$$

38 $$\begin{aligned} u_{17}\left( x,t\right)= & {} {\frac{8\,\sigma +\alpha _{{2}}}{2\alpha _{{3}}}} + {\frac{{6}}{\alpha _{{3}}}} \bigg ({-\sqrt{\sigma }\cot \left( \sqrt{\sigma }\xi \right) } \bigg )^{2} + {\frac{{6\sigma }^{2}}{\alpha _{{3}}}}\bigg ({-\sqrt{\sigma }\cot \left( \sqrt{\sigma }\xi \right) } \bigg )^{-2}, \end{aligned}$$

where $$\xi =x-{ \left( -\,{\frac{\alpha _{{1}} \left( 20\,\sigma +\alpha _{{2}} \right) }{2\alpha _{{3}}}}\right) t}$$.

**Family-3. When** $\sigma =0$, **then the possible solutions are**

39 $$\begin{aligned} u_{18}\left( x,t\right) ={\frac{\alpha _{{2}}}{2\alpha _{{3}}}} + {\frac{{6}}{\alpha _{{3}}}} (-\frac{1}{\xi })^{2}, \end{aligned}$$

where $$\xi =x-{ \left( -{\frac{\alpha _{{1}} \alpha _{{2}} }{2\alpha _{{3}}}}\right) t}$$.

**Case 5**. Plugging Eq. (21) along with Eq. (15) into Eq. (13), then solitary waves solutions of Eq. (12) can be expressed in form of following families:

**Family-1. When** $\sigma <0$, **then the possible solutions are**

40 $$\begin{aligned} u_{19}\left( x,t\right)= & {} \dfrac{1}{2}\,{\frac{ \alpha _{{2}}\pm \sqrt{{\alpha _{{2}}}^{2}+8\,\alpha _{{3}}\lambda _{{2}}}}{\alpha _{{3}}}} + \,{\frac{{6\,\sigma }^{2}}{\alpha _{{3}}}} \bigg ({-\sqrt{-\sigma }\tanh \left( \sqrt{-\sigma }\xi \right) } \bigg )^{-2}, \end{aligned}$$

41 $$\begin{aligned} u_{20}\left( x,t\right)= & {} \dfrac{1}{2}\,{\frac{ \alpha _{{2}}\pm \sqrt{{\alpha _{{2}}}^{2}+8\,\alpha _{{3}}\lambda _{{2}}}}{\alpha _{{3}}}} + {\frac{{6\,\sigma }^{2}}{\alpha _{{3}}}} \bigg ({-\sqrt{-\sigma }\coth \left( \sqrt{-\sigma }\xi \right) } \bigg )^{-2}, \end{aligned}$$

where $$\xi =x-{\left( \dfrac{1}{2}\,{\frac{\alpha _{{1}} \left( \alpha _{{2}}+\sqrt{{\alpha _{{2}}} ^{2}+48\,{\sigma }^{2}} \right) }{\alpha _{{3}}}}\right) t}$$.

**Family-2. When** $\sigma >0$, **then the possible solutions are**

42 $$\begin{aligned} u_{21}\left( x,t\right)= & {} \dfrac{1}{2}\,{\frac{ \alpha _{{2}}\pm \sqrt{{\alpha _{{2}}}^{2}+8\,\alpha _{{3}}\lambda _{{2}}}}{\alpha _{{3}}}} + {\frac{{6\,\sigma }^{2}}{\alpha _{{3}}}} \bigg (\sqrt{\sigma }\tan \left( \sqrt{\sigma }\xi \right) \bigg )^{-2}, \end{aligned}$$

43 $$\begin{aligned} u_{22}\left( x,t\right)= & {} \dfrac{1}{2}\,{\frac{ \alpha _{{2}}\pm \sqrt{{\alpha _{{2}}}^{2}+8\,\alpha _{{3}}\lambda _{{2}}}}{\alpha _{{3}}}} +{\frac{{6\,\sigma }^{2}}{\alpha _{{3}}}} \bigg (-\sqrt{\sigma }\cot \left( \sqrt{\sigma }\xi \right) \bigg )^{-2}. \end{aligned}$$

where $$\xi =x-{ \left( \dfrac{1}{2}\,{\frac{\alpha _{{1}} \left( \alpha _{{2}}+\sqrt{{\alpha _{{2}}} ^{2}+48\,{\sigma }^{2}} \right) }{\alpha _{{3}}}}\right) t}$$.

## The graphical representation

This section provides the graphical representations that explain the outcomes of our study, see Figs. 1, 2, 3, 4, 5, 6, 7, 8, 9, 10, 11, 12, 13, 14, 15, 16, 17, 18 and 19.

**Figure 1:**
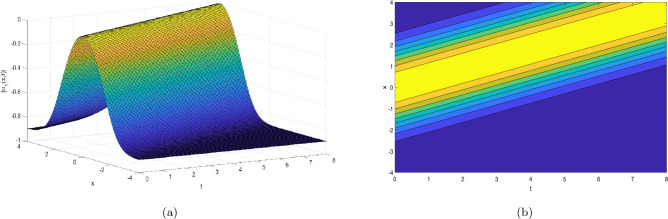
Take $\alpha _{1} = 1.7,\alpha _{2} = 1.2, \alpha _{3} = 1.5, c = 0.45,\sigma = - 0.007$ for 3D and contour plots of Eq. (22).

**Figure 2:**
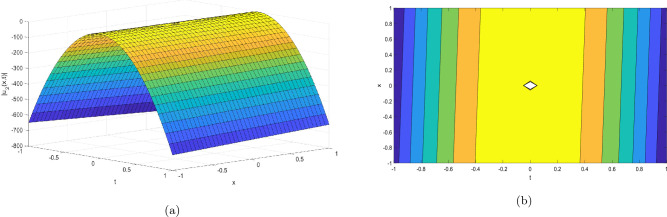
Take $\alpha _{1} = 5.5,\alpha _{2} = 5.25, \alpha _{3} =20.05, c = 50.75,\sigma = -2.09$ for 3D and contour plots of Eq. (23).

**Figure 3:**
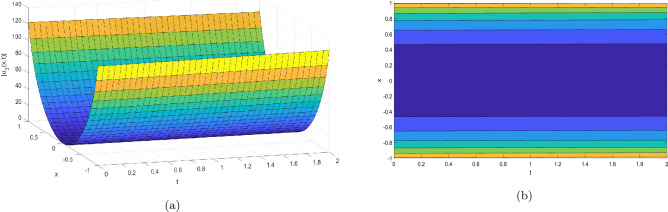
Take $\alpha _{1} = 2.07,\alpha _{2} = 0.02, \alpha _{3} =0.05, c = 0.004,\sigma = 0.75$ for 3D and contour plots of Eq. (24).

**Figure 4:**
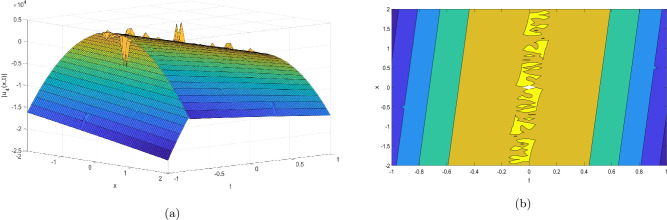
Take $\alpha _{1} = 15.5,\alpha _{2} = 10.25, \alpha _{3} =40.05, c = 25.75, \sigma = 30.9$ for 3D and contour plots of Eq. (25).

**Figure 5:**
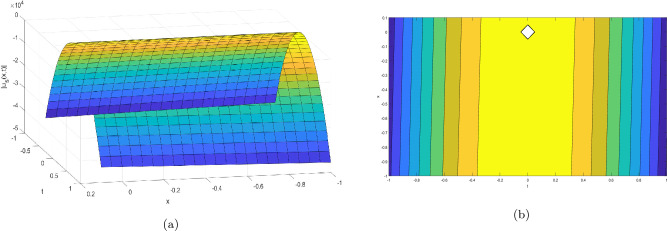
Take $\alpha _{1} = 15.5,\alpha _{2} = 20.25, \alpha _{3} =20.05, c = 40.75, \sigma = 20.9$ for 3D and contour plots of Eq. (26).

**Figure 6:**
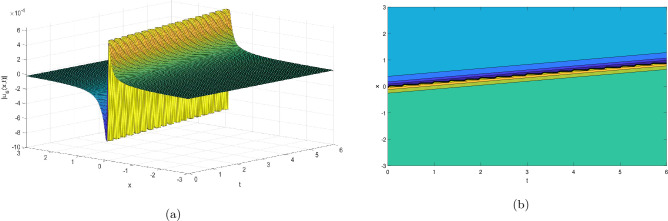
Take $\alpha _{1} = 1.7, \alpha _{3} =1.5, c = 0.15, \sigma = -0.009$ for 3D and contour plots of Eq. (27).

**Figure 7:**
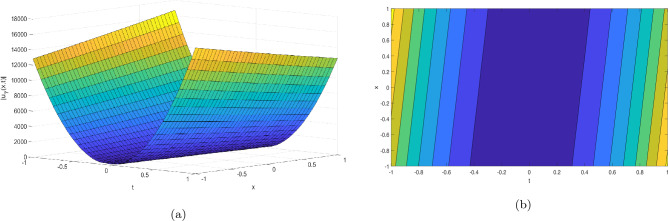
Take $\alpha _{1} = 2.7, \alpha _{3} =2.5, c = 15.15, \sigma = -8.007$ for 3D and contour plots of Eq. (28).

**Figure 8:**
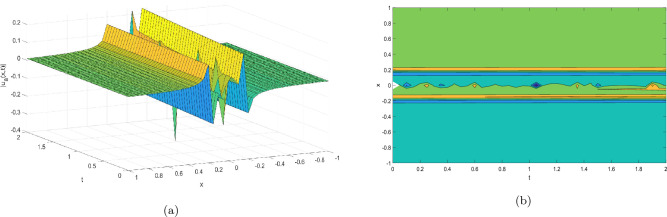
Take $\alpha _{1} = 1.007, \alpha _{3} =1.005, c = 0.0015, \sigma = 0.007$ for 3D and contour plots of Eq. (29).

**Figure 9:**
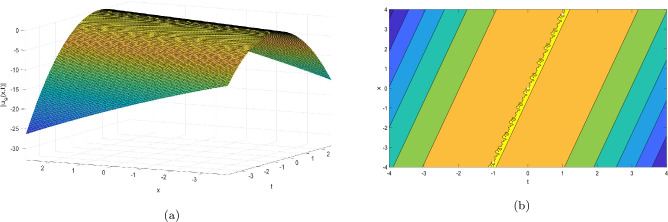
Take $\alpha _{1} = 0.05, \alpha _{2}= =0.025, \alpha _{3}= 0.005, c = 3.75, \sigma = 0.009$ for 3D and contour plots of Eq. (30).

**Figure 10:**
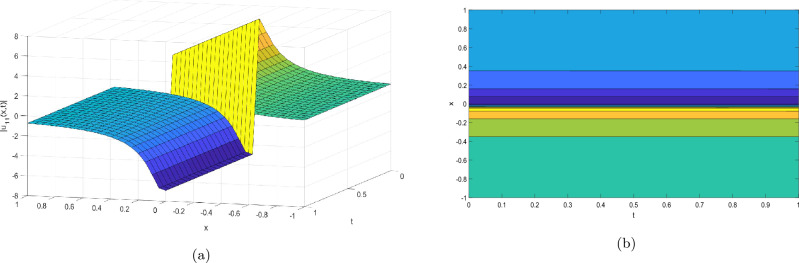
Take $\alpha _{1} =-0.03, \alpha _{2}= =2.076, \alpha _{3}= 5.085, \sigma = -0.0109$ for 3D and contour plots of Eq. (31).

**Figure 11:**
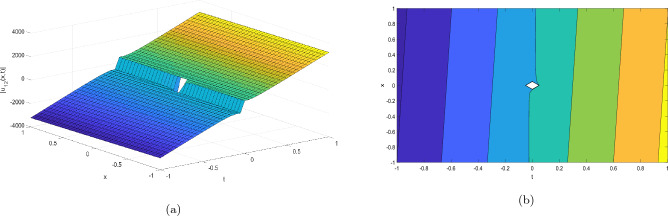
Take $\alpha _{1} =1.107, \alpha _{2}= =15.7, \alpha _{3}= 4.5, \sigma = -18.007$ for 3D and contour plots of Eq. (32).

**Figure 12:**
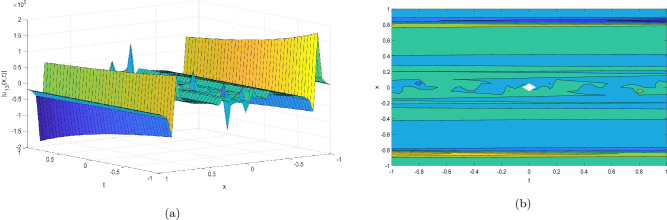
Take $\alpha _{1} =0.007, \alpha _{2}= =10.5, \alpha _{3}= -10.5, \sigma = 1.7$ for 3D and contour plots of Eq.(33).

**Figure 13:**
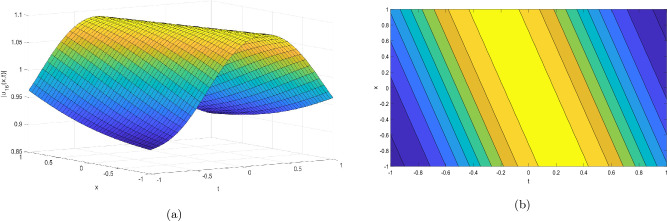
Take $\alpha _{1} =3.7, \alpha _{2}= =5.5, \alpha _{3}= -2.5, \sigma = -0.007$ for 3D and contour plots of Eq. (35).

**Figure 14:**
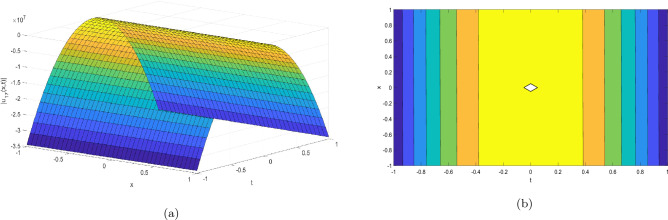
Take $\alpha _{1} =-11.7, \alpha _{2}= =0.5, \alpha _{3}= 0.5, \sigma = -2.7$ for 3D and contour plots of Eq. (36).

**Figure 15:**
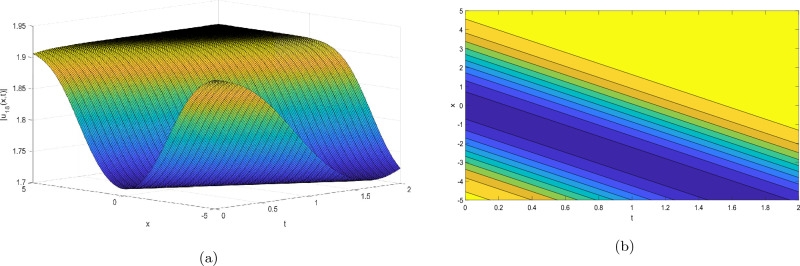
Take $\alpha _{1} =-1.7, \alpha _{2}= =8.5, \alpha _{3}= 2.5, \sigma = 0.007$ for 3D and contour plots of Eq. (37).

**Figure 16:**
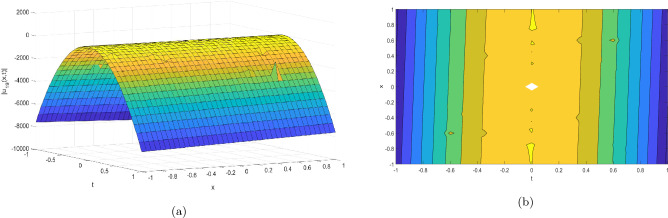
Take $\alpha _{1} =-20.7, \alpha _{2}= =10.5, \alpha _{3}= 13.5, \sigma = 2.7$ for 3D and contour plots of Eq. (38).

**Figure 17:**
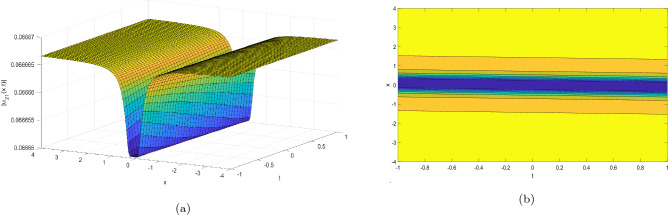
Take $\alpha _{1} =0.7, \alpha _{2}= =0.2, \alpha _{3}= 1.5, \alpha _{4}= 0.5, \sigma = -0.007$ for 3D and contour plots of Eq. (40).

**Figure 18:**
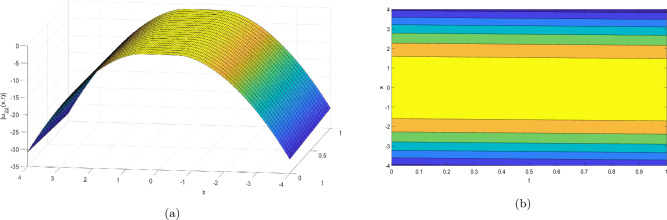
Take $\alpha _{1} =0.07, \alpha _{2}= =0.22, \alpha _{3}= 1.52, \alpha _{4}= 0.5, \sigma = -0.7$ for 3D and contour plots of Eq. (41).

**Figure 19:**
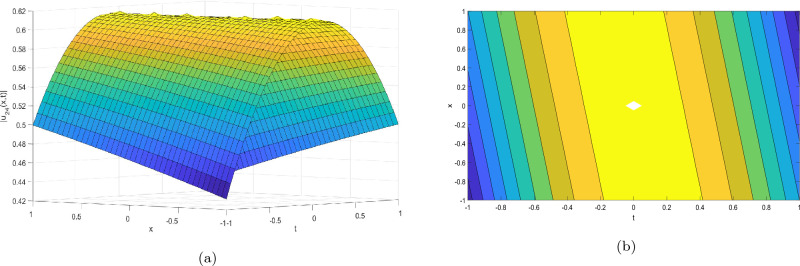
Take $\alpha _{1} =10.07, \alpha _{2}= =12.22, \alpha _{3}= 14.52, \alpha _{4}= 20.5, \sigma = 0.07$ for 3D and contour plots of Eq. (43).

## Discussion and conclusion

In order to visualize the physical behavior of the solutions and explain the shape of solitary waves, this part displays the solutions derived using 3D and contour graphs. The wave height is shown in 3D graphs as a function of horizontal location, vertical position, and time, allowing us to view the wave propagation in three dimensions.We can learn more about the shape, amplitude, speed, and direction of solitary waves by viewing these plots.

Contrarily, contour plots show how wave height varies spatially as a function of horizontal and vertical coordinates, with curves indicating regions of constant height. These plots offer a thorough and quantitative depiction of the wave behavior and properties throughout time, enabling us to examine its shape, amplitude, and speed. Overall,3D and contour plots are useful tools for research and understanding of solitary wave solutions.

The Figs. 1, 6, 10, 13 and 17 display dark solitary wave solutions while Figs. 2, 7, 11, 14 and 18 display singular dark solitary wave solutions. Similarly the Figs. 3, 4, 8, 9, 12, 15, 16 and 19 display periodic solitary wave solutions. Furthermore the Fig. 5 display rational solitary wave solutions.

This research article introduced the modified extended direct algebraic method as a powerful tool for discovering new types of solitary wave solutions for the nonlinear Murray equation in mathematical biology. The study demonstrated that this method consistently produces exact and stable solutions across a wide range of parameters. These newly identified solitary wave solutions have significant implications for simulating various biological systems, such as blood flow and tumor growth. Moreover, they contribute to the refinement of mathematical models in the field of mathematical biology. Future research avenues may explore the dynamical and physical characteristics of these recently discovered solitary wave solutions and investigate their potential applications in diverse branches of engineering and science. In conclusion, this study provides valuable insights into the behavior of solitary waves within biological systems, paving the way for further exploration and practical applications in this promising field.

## Data Availability

The datasets used and/or analysed during the current study available from the corresponding author on reasonable request.
